# PSA Based Biomarkers, Imagistic Techniques and Combined Tests for a Better Diagnostic of Localized Prostate Cancer

**DOI:** 10.3390/diagnostics10100806

**Published:** 2020-10-10

**Authors:** Vlad Cristian Munteanu, Raluca Andrada Munteanu, Diana Gulei, Vlad Horia Schitcu, Bogdan Petrut, Ioana Berindan Neagoe, Patriciu Achimas Cadariu, Ioan Coman

**Affiliations:** 1 Department of Urology, The Oncology Institute “Prof Dr. Ion Chiricuta”, 400015 Cluj-Napoca, Romania; schitcu@yahoo.com (V.H.S.); bogdan.petrut@gmail.com (B.P.); 2 Department of Urology, “Iuliu Hatieganu” University of Medicine and Pharmacy, 400012 Cluj-Napoca, Romania; 3 MedFuture—Research Center for Advanced Medicine, “Iuliu Hatieganu” University of Medicine and Pharmacy, 400337 Cluj-Napoca, Romania; muresan.raluca.andrada@gmail.com (R.A.M.); diana.c.gulei@gmail.com (D.G.); 4 Research Center for Functional Genomics, Biomedicine and Translational Medicine, “Iuliu Hatieganu” University of Medicine and Pharmacy, 400337 Cluj-Napoca, Romania; ioananeagoe29@gmail.com; 5 Department of Functional Genomics and Experimental Pathology, The Oncology Institute “Prof. Dr. Ion Chiricuta”, 400015 Cluj-Napoca, Romania; 6 Surgery Department, The Oncology Institute “Prof. Dr. Ion Chiricuţă”, 400015 Cluj-Napoca, Romania; Pachimas@umfcluj.ro; 7 Department of Surgery and Gynecological Oncology, the University of Medicine and Pharmacy “Iuliu Hatieganu”, 400337 Cluj-Napoca, Romania; 8 Department of Urology, Clinical Municipal Hospital, 400139 Cluj-Napoca, Romania

**Keywords:** prostate cancer, biomarkers, screening, prostate-specific antigen, magnetic resonance imaging, prostate health index, 4Kscore, Stockholm 3 test, multiparametric ultrasound

## Abstract

Prostate cancer represents the most encountered urinary malignancy in males over 50 years old, and the second most diagnosed after lung cancer globally. Digital rectal examination and prostatic specific antigen were the long-time standard tools for diagnosis but with a significant risk of overdiagnosis and overtreatment. Magnetic resonance imaging recently entered the diagnosis process, but to this date, there is no specific biomarker that accurately indicates whether to proceed with the prostate biopsy. Research in this area has gone towards this direction, and recently, serum, urine, imagistic, tissue biomarkers, and Risk Calculators promise to help better diagnose and stratify prostate cancer. In order to eliminate the comorbidities that appear along with the diagnosis and treatment of this disease, there is a constant need to implement new diagnostic strategies. Important uro-oncology associations recommend the use of novel biomarkers in the grey area of prostate cancer, to better distinguish the next step in the diagnostic process. Although it is not that simple, they should be integrated according to the clinical policies, and it should be considered that statistical significance does not always equal clinical significance. In this review, we analyzed the contribution of prostate-specific antigen (PSA)-based biomarkers (PHI, PHID, 4Kscore, STHLM3), imagistic techniques (mp-MRI and mp-US), and combined tests in the early diagnosis process of localized prostate cancer.

## 1. Introduction

Biomarkers indicate the presence of a disease, and ideally, they should be identified through non-invasive and inexpensive tests. Specificity and sensitivity are important in order to accurately differentiate malign from the benign process and distinguish between indolent and aggressive tumors. In consequence, the use of these tests should help physicians confirm cancers, diagnose them early, evaluate the risk of progression or recurrence, and could also monitor the effectiveness of treatment [1].

Prostate cancer (PCa) is the second most common cancer among men, and globally, it represents 13.5% of all cancers, and it occupies the fifth place in terms of mortality [2,3]. In 2018, 1.3 million new patients received the diagnosis of prostate cancer, and 359,000 died from this disease [2,4]. It is age-specific, men with ages above 45–50 years being at risk, but it also presents a genetic predisposition, for example, African and Caribbean ethnic groups being at high risk compared to other ethnic groups [5].

Even though direct rectal examination (DRE) was the first method of screening PCa, there is still much debate about its usefulness. As reflected in the European Association of Urology (EAU) Guidelines in 2020, the sensitivity and specificity of DRE in the primary care settings is below 60% and is not recommended for the exclusion of PCa [6].

The Food and Drug Administration (FDA) first approved the usage of prostate-specific antigen (PSA) in PCa in 1986, and this revolutionized the diagnosis process of PCa, and allowed the detection in earlier stages, in contrast to previously used techniques where most of the cases were diagnosed in locally advanced or metastases stages. This tool helped physicians improve prostate cancer screening and consequently reduce the PCa specific mortality [7].

At this moment, systematic biopsy remains the gold standard in PCa diagnosis, as stated by EAU Guidelines from 2020 with a 1b level of evidence (evidence obtained from at least one randomized trial) [8].

Once the diagnosis has been established, some patients have the option for active surveillance [9] while others, with more aggressive cancers, need active treatment (radical prostatectomy or radiation therapy) [10], both with short- and long-term complications and changes in quality of life. The perioperative complications at 30 days were 21% for the patients that underwent radical prostatectomy; the long-term complications were urinary incontinence in 17% of cases, erectile dysfunction in 81% of cases, and bowel dysfunction in 12% of cases [11]. Up to 47% of men presented a degree of incontinence at 6 months after surgery, which downgraded to 18% at 6 years, erectile function decreased by 50% for potent men at 6 months after treatment and did not recover over time [12]. Of the patients, 65% presented severe erectile dysfunction, and digestive complications (constipation, diarrhea, appetite loss), cognitive, emotional, and functional scores were worse for treated patients compared to controls. [13].

Diagnosing and treating based only on PSA can lead to overdiagnosis and overtreatment of tumors that are not clinically significant [14,15]. Along with the comorbidities that come with those, many researchers started to focus on new biomarkers for diagnosing and staging PCa, evaluating them from the blood (PHI, 4Kscore, S3M), from urine (PCA3, TMPRSS2-ERG, TDRD1, DLX1, HOX6), or through radiographic modalities (multiparametric magnetic resonance imaging(mpMRI), multiparametric ultrasound (mpUS) [14,15]. Risk calculators have been developed, they combine age, PSA, DRE, trans-rectal ultrasound (TRUS), PSA density (PSAD), prostate volume, and more recent MRIs to better differentiate indolent cancers from clinically significant ones, but they can be improved [16,17].

In this review, we aim to discuss the positive value brought by PSA-derived biomarkers, MRI, US, and the combinations of these methods for a better early diagnosis process of localized PCa, with a PSA level under 10 ng/mL. We will explain how to interpret all these biomarkers and how they could be applied in day-to-day clinical practice in the screening and diagnosis process, in the active surveillance process, and in scenarios with previous negative biopsies. These tools combined should offer a better risk stratification of PCa. The chosen assays and imaging techniques are non-invasive tests with high sensitivity and specificity for diagnosing PCa, they can help discriminate between benign and aggressive cancers.

## 2. Classic PCa Diagnosis Pathways

### 2.1. DRE (Digital Rectal Examination)

DRE is not recommended routinely as a tool in PCa primary care, which is a factor that could contribute to unnecessary biopsies, being a consequence that leads to overdiagnosis and overtreatment [18]. Despite this aspect, it is suggested that DRE has not lost its value completely. For example, patients with PSA levels under 4 ng/mL DRE had a sensitivity rate of 35%. In these cases, DRE represents a tool that should be used in the clinical evaluation of men with PCa under Active Surveillance (AS) regardless of PSA values, even though DRE alone cannot rule out the presence of clinically significant PCa (csPCa). Men under AS with an initial DRE negative, which becomes positive overtime, should be closely monitored [19].

Abnormal DRE is not universally defined, it is a subjective maneuver, and it does not present any technical aspects. For example, in a Canadian survey, half of the graduating students, during their clerkship training, had never performed DRE, only half of the primary care physicians felt confident in detecting nodules through DRE [18].

DREs could help in deciding whether to rebiopsy patients on screening programs. If DRE is normal, the rebiopsy could be postponed until a later screening meeting; this can reduce the risk of diagnosing indolent cancers and can reduce the number of unnecessary biopsies [20].

In the ERSPC (European Randomized Study of Screening for Prostate Cancer) trial, 15% of DREs results were considered false positive because they could not be confirmed on biopsy [21].

### 2.2. PSA (Prostate-Specific Antigen) and fPSA (freePSA)

Secreted by the prostatic epithelium, PSA is organ-specific, not cancer-specific, which means that other pathologies also influence PSA levels, such as prostatitis or benign prostatic hyperplasia (BPH), androgen levels, DRE, body mass index (BMI) (hemodilution of PSA), prostatic trauma (biopsy), urinary retention, ejaculation under 24 h [7].

A PSA level above 4 ng/mL is considered suspect for PCa, although levels between 4–10 ng/mL are considered to be in a grey zone. Due to this particularity concerning PSA, overdiagnosis and overtreatment are risks, with severe and unnecessary complications [1]. Evidence shows that up to 25% of patients with normal PSA values can have underlying PCa [5].

All these data show that PSA alone is not a good predictor of biopsy results, and many studies suggest that it should be accompanied by other biomarkers to improve the outcome [22,23].

Some authors are recommending precaution when using PSA screening for PCa [24]. The argument states that it is more likely that patients will suffer because of the biopsy or radical treatment complications. Screening data from major randomized controlled trials like The Cluster Randomized Trial of PSA Testing for Prostate Cancer (CAP), The European Randomised Study of Screening for Prostate Cancer (ERSPC), The Prostate, Lung, Colorectal and Ovarian (PLCO), with a screening group and a control group, over 10 years, showed that for every 1000 people, two patients from the screening group will die of PCa. Three patients from the non-screening group will have the same outcome. From the first cohort, 94 patients presented blood in semen, 45 experienced pain, 19 fever, 67 blood in urine, and one sepsis, all being complications of prostate biopsy. Moreover, from the treated group, 25 patients had erectile dysfunction, without the possibility of penetration, and three presented urinary incontinence due to radical treatment. In the screening group, PSA screening helps diagnose more cancers at any stage (18 more/1000 patients) than in the non-screening group and identifies more localized cancers (14 more/1000 patients). This small benefit brought by PSA can be associated with short or long-term complications [24].

For men who underwent prostate biopsies for risk of PCa, the 30 days complications rates were 17%, of which 8.3% were non-sepsis genito-urinary infections, 7.3% with bleeding complications, and 2.9% presented urinary retention. Previous use of fluoroquinolones and anticoagulants, age above 70, previous cancer diagnoses were associated with a higher risk of complications sepsis or hospitalization [25]. Other authors report a 1.5% risk of infection, 12.5% hematuria, 3.6% hematospermia, and 7% pain after prostate biopsy [26]. When comparing the complication rates between transrectal (TR) and transperineal (TP) prostate biopsies, the urosepsis rate was 2.46% vs. 0.4%, urinary retention 2.76% vs. 3.63%, hematuria 0.23% vs. 0.81% [27].

Schröder FH et al., 2014 determined that long-term screening for PCa can reduce PCa mortality by about 9% [25]. To prevent one death from PCa, 1410 men needed to be screened, and 48 of them had to be treated [21]. In the same trial, 10.4% of PSAs resulted in false-positive results on biopsy, but other studies report up to 75% [21].

Even though it is not recommended to only use the PSA value for PCa diagnosis, to date, it is the most useful tool for follow-up after active treatment [1]. It will continue to be in the spotlight for prostate cancer because it has implications in angiogenesis, invasion, metastasis, and cancer signaling [28].

A large percentage of PSA is bound to α1-antichymotrypsin, α2-macroglobulin, and α1-proteinase inhibitor, in a proportion of about 85%, the rest is represented as freePSA (fPSA) [1]. The freePSA ratio (fPSA%) is fPSA/tPSA × 100. Usually, a high PSA and a low fPSA% is associated with more aggressive PCas. FreePSA is also used as a component of PHI score and 4Kscore [1].

A computer model was created based on the data of the PLCO trial, the area under the curve (AUC) was calculated for the PCa diagnostic prediction. PSA showed an AUC of 0.63, freePSA an AUC of 0.50, free to total ratio an AUC 0.65, and the computer model an AUC of 0.72. FreePSA alone is a poor diagnosis tool, but integrated into other tests (PHI, 4Kscore), it raises their accuracy [29].

In Table 1, the prostate cancer screening indications offered by AUA, EAU, ESMO, ACS based on PSA are highlighted.

### 2.3. PSA Density (PSAD)

PSAD is defined as the PSA value divided by the volume of the prostate, with thresholds between 0.08 ng/mL/cc and 0.15 ng/mL/cc, it can be helpful in making biopsy decisions, when the PSA <10 ng/mL. It was compared with PSA in terms of diagnosing PCa, and proved to be significantly superior (*p* = 0.011) [33,34]. Combined with PSA, fPSA, or even with mpMRI, it can help better discriminate whether to proceed with the prostate biopsy [35]. Adding PSAD in this landscape can lead to better discrimination of PCa than PSA alone (AUC 0.75 vs. 0.73, *p* < 0.05) [36].

### 2.4. MpMRI (Multiparametric Magnetic Resonance imaging)

Pre-biopsy MRI is now part of the EAU guidelines for biopsy naive patients [8] even though some authors advise against it, contesting the idea that systematic TRUS biopsy would miss many high-grade PCas. According to Kasivisvanathan et al., in the PRECISION study in 2018, MRI increased the diagnosis of Gleason grade (GG) 5 PCa by 2.8%. The contesting authors interpreted this as about 10,000 cases missed by TRUS per year, leading to an increased number of metastatic cancers, but that is not the present case [37]. Pre-biopsy MRI with targeted biopsies does increase the risk of over-diagnosing. Still, it should also be interpreted according to tumor size, coefficient of diffusion, PSA density, and systematic biopsy results [37].

## 3. Non-Invasive Biomarkers in PCa Diagnosis

Below, we will detail the advantages of these biomarkers over PSA in the screening and diagnosis process, in monitoring patients on AS, in scenarios with previous negative biopsies, as well as their power to differentiate between indolent and aggressive tumors. The biomarkers detailed below were chosen because they all contain the PSA component and PSA isoforms, and they can be easily implemented in clinical practice.

### 3.1. Prostate Health Index (PHI) and Prostate Health Index Density (PHID)

PHI is a blood-based diagnostic test that combines PSA, freePSA, and [-2]pro-PSA (precursor of PSA) values. The test was validated, especially for diagnosing clinically significant cancers for patients with a PSA of 4–10 ng/mL. The utility of the test is that it can help reduce unnecessary biopsies, and it proved higher specificity and sensitivity than total PSA (tPSA) and free PSA% [1], with cutoff values that can range between 20–40 [38,39]. PHID is defined as PHI divided by prostate volume with a cutoff above 0.9 [34].

Eveline A M Heijnsdijk et al. in 2016 created a microsimulation based on the ERSPC trial results, which evaluated the effects of PHI added to the PSA screening. They found that using PHI could have reduced the number of negative biopsies by 23% at a PSA level 3–10 ng/mL, but using a cutoff value of 35%, it can decrease the biopsies number by 42% [40].

Taken together, PHI and PHID proved to be better than PSA, fPSA% and PSAD when deciding which patients should undergo biopsy and which should not. For all cancers diagnosed, the AUC was 0.7222 and 0.739 for PHI and PHID, but the AUCs for PSA, fPSA%, and PSAD were 0.595, 0.612, and 0.698. When focusing on clinically significant cancers, the receiver operating characteristics (ROC) for PHI and PHID were 0.757 and 0.764, but for PSA, fPSA% and PSAD, they were only 0.635, 0.627, and 0.732. For all cancers and significant cancers, the differences between PHI, PHID vs. PSA, fPSA%, were statistically significant, but not statistically significant when PHI and PHID were compared with PSAD. With a cutoff value of >40 for PHI and >0.9 for PHID, these two tests excluded the need for biopsy for 20% of patients and only missed 1 Gleason 8 PCa, which after prostatectomy downgraded to Gleason 7(4 + 3), pT2a [34].

In the multicentric study, PRIM (PHI to refIne MRI), conducted by Lois Kim et al. 2020 on a cohort of 545 men, PHI proved better detection of significant (GG ≥ 2) PCa than PSA and PSAD with an AUC of 0.82 vs. 0.70, 0.79. When mpMRI was negative, PHI showed an AUC of 0.78 vs. 0.64 and 0.76 (PSA and PSAD) in detecting PCa GG ≥ 2. For PHI ≥ 20, the negative predictive value (NPV) was 0.85 with 1.1% missed cancers, and for PHI ≥ 30 the NPV was 0.9 with 7.7% missed cancers. When using the PHI ≥ 30, and considering GG ≥ 2 as an endpoint, PHI managed to reduce the number of mpMRIs by 35% with 9% missed cancers and reduced the unnecessary biopsies by 40% with 8% missed tumors [41].

Men on active surveillance can benefit from PHI and PHID, which can help reclassify the tumor grade. Patients with higher PHI values had a higher chance of reclassification. A PHI score under 25.6 and a Prostate Imaging-Reporting and Data System (PI-RADS)v2 lesions ≤3, could help avoid 20% of prostate biopsies while missing only 2.6% that would have been reclassified, but if MRI examination is negative, as many as 41% of biopsies could be bypassed while missing 11% of tumors that would have been reclassified [42].

Men with PCa can be identified and separated from healthy men using PHI (AUC 0.887). Still, for differentiating prostate cancer patients from patients with BPH, p2PSA showed better results than PHI (AUC 0.7333 vs. 0.639). As the tumor becomes more aggressive and the disease is more advanced, the level of PHI increases, and if there are positive margins, it remains high even after radical prostatectomy. PHI proved to be an independent predictor for diagnosis and prognosis [43].

PHI is a PSA-based blood marker that showed its usefulness in biopsy naïve patients, saving a lot of them from unnecessary biopsies and possible biopsy complications. It can differentiate PCa patients from healthy ones and patients with BPH. If the mpMRI is negative, it helps urologists decide whether to biopsy or not and if it is performed before the MRI test, it can also help avoid it, a decision which also has economic implications. Active surveillance patients can also benefit from the PHI test in the decision to undergo rebiopsy or not. With all the benefits that it has shown, large randomized studies are needed to establish its definitive role.

### 3.2. 4 Kscore (4 Kallikreins Score)

The 4kscore (4K) is associated with the test results of four kallikreins: totalPSA, freePSA, intactPSA (isoform of freePSA), and human kallikrein 2 (hk2). The results correspond to one of the three risk categories: low risk at 1–7.5%, moderate risk at 7.5–19%, and above 20% is considered high risk for the positive disease at biopsy. It can be used for patients with previous negative biopsy or patients with an indication for prostate biopsy. At a PSA >3 ng/mL, the 4Kscore is more sensitive for high-grade cancer detection than clinical variables alone. For PHI and 4Kscore, the diagnosis performance was similar, but they each outperformed PSA age stratification for the high-grade cancer prediction [1].

For a cohort of 1012 Caucasian men undergoing a prostate biopsy, a 4Kscore spares 252 biopsies, while missing 19 of 195 aggressive PCa detected on biopsy. In the same study, the 4Kscore showed better results than PSA for reclassifying specimens at first biopsy (AUC 0.78 vs. 0.74), but not at subsequent ones (AUC 0.75 vs. 0.76) [44].

Using serum samples from the ERSPC clinical trial, the Rotterdam section, Andrew J. Vickers et al. 2010 calculated the 4Kscore, and found that it reduces the number of biopsies by 36%, postponing the diagnosis of 43 low-grade PCa and 4 high-grade PCa from a total of 1000 biopsies. The base model (DRE, PSA, and age) versus the full model (age, PSA, freePSA, and hk2) showed an AUC of 0.585 versus 0.711 for diagnosing any cancer, and AUC 0.709 vs. 0.798 in detecting high-grade cancer. Biopsy based on the kallikrein model leads to superior clinical outcomes. In this setting, adding PSA velocity did not improve the biopsy outcome [23].

In the same clinical trial, but at rebiopsy (the first biopsy was negative), the AUC for 4Kscore vs. base model (PSA, age, DRE) was 0.681 vs. 0.584 for detection of any PCa, and 0.873 vs. 0.764 in detecting high-grade cancer. In this context, the authors believe that by applying the 4Kscore, it is possible to reduce biopsies by 82% (risk cancer of 20%) while postponing the diagnosis for 64 low-grade PCa and 3 high-grade PCa (none Gleason ≥ 8) per 1000 patients [22].

At Skane University Hospital, between 2004 and 2010, a screening that included 749 men with PSA levels ≥3.0 ng/mL, fPSA% ≤20%, or abnormal DRE underwent biopsy. The authors created a 4K model with higher discrimination than the classic model that included PSA, age, and DRE. It better predicted high-risk PCa, with an AUC of 0.777 for the 4Kscore, and only AUC: 0.719 for PSA and age. When the model was adjusted for age, 4K, and DRE, the AUC raised to 0.784. This model could reduce unnecessary biopsies by about 25%, and miss about 6% of high-grade cancers [45].

In a multiethnic group (African Americans, Japanese, Latinos, Native Hawaiians, and Whites) divided into controls and disease subsets, the 4Kscore was compared with PSA+fPSA and PSA alone. It showed an AUC of 0.748 vs. 0.7111 and 0.669 for any given PCa. When evaluating these tests for the accuracy of finding aggressive PCa, the AUC results were 0.782 vs. 0.739 and 0.685. This prospective study showed the 4Kscore′s accuracy to discriminate benign from malignant cases and that it can be superior to PSA and fPSA in diagnosing aggressive and non-aggressive tumors [46].

The Rotterdam Prostate Cancer Risk Calculator (RPCRC) was compared with the 4Kscore, and they both showed similar AUCs (0.88 vs. 0.87), but combined, they improved the AUC to 0.89, which is statistically better than each test alone. The tests used either independently or synergistically could reduce the number of unnecessary biopsies by 65–66% while missing 14–16% of csPCas [47].

The 4Kscore showed superiority over PSA, fPSA, PSA+fPSA, DRE in terms of diagnosis indolent and aggressive PCa, and specimen reclassification. It can be used in cases of high suspicion PCa with previous negative biopsies, and it also showed superiority over a Risk Calculator. Still, the two together had the best outcome in detecting PCas.

### 3.3. The Stockholm-3 Model for Prostate Cancer Detection (STHLM3)

The STHLM3 model or S3M model consists of a combination of blood biomarkers (free PSA, PSA, intact PSA, MSMB (microseminoprotein beta), MIC1 (macrophage inhibitory cytokine-1), hk2), genetic polymorphisms (232 SNPs) and clinical details such as age, previous biopsies or prostate exams [48]. This model proved to be more sensitive in diagnosing PCa than PSA for men between the age of 50–70 [14,49]. It can serve as a biomarker for high-risk cohorts such as African Americans (AUC 0.852), and Hispanic Caucasians (AUC 0.895) [48].

Between 2012 and 2015, 59,149 participants with ages between 50–69 underwent PSA and S3M testing in the STHML3 study. Patients with PSA levels above 3 ng/mL and S3M suggestive for Gleason 7 underwent biopsy (7416 men). The S3M test showed an AUC of 0.75 compared to PSA (AUC 0.58). The authors concluded that S3M could reduce unnecessary biopsies up to 34% without the risk of missing any GS ≥ 7 tumors [50].

Performing MRI at an S3M risk above 10% could reduce the number of MRIs and prostate biopsies by 38%, and it could diagnose 42% less insignificant PCa and miss 8% of csPCa [51]. The S3M-MRI prediction model proved to be superior in predicting an International Society of Urological pathology (ISUP) grade >2 PCa than the S3M model alone or only MRI (AUC 0.88 vs. 0.86 vs. 0.83) [52].

This test vas validated on a cohort of 60,000 men, and it showed very good results in separating indolent from clinically significant tumors, and it showed superiority to PSA in the PCa diagnosing process. It is clinically available only in Denmark, Norway, Sweden and Finland but the downside is that it is available only in these countries.

## 4. Imagistic Techniques

MRI is already integrated into most guidelines concerning PCa diagnosis. This examination is recommended before the biopsy, in the case of abnormal PSA value or abnormal DRE [8]. The US is an essential tool that every urologist should know how to handle. It is low cost, non-invasive, and it can be used in a variety of actions, from a simple consultation to post-surgery follow-up. The technique advanced and, nowadays, it can be used in the diagnosis process of PCa.

### 4.1. Mp-MRI (Multiparametric Magnetic Resonance Imaging)

This can increase detection, especially for anterior lesion, and it can accurately measure the prostate volume, tumor volume, aggressiveness, invasion of the capsule, or neurovascular bundles. Mp-MRI combines T2 imaging, apparent diffusion coefficient (ADC) calculations, dynamic contrast enhancement (DCE), diffusion sequences, and sometimes spectral MRI. It presents high sensitivity for tumors above 1 cm^3^, Gleason grade above 1, but for smaller lesions, or lower grades, it is limited [53]. In contrast, bi-parametric MRI (bp-MRI) uses only T2 weighted image (T2W), diffusion-weighted imaging (DWI), and ADC, lowering the investigation time from 27 min for mp-MRI to just 17 min for bp-MRI, and being equally good in detecting PCa. Bp-MRI vs. mp-MRI presented a sensitivity of 0.94–0.96 vs. 0.93–1.00 and specificity of 0.15 vs. 0.04–0.16 [53].

In the PRECISION study, Kasivisvanathan et al., in 2018, randomized 500 patients into a 1:1 (252 MRI TB (targeted biopsy) group and 248 into a systematic biopsy (SB) group. CsPCa was detected in 95 patients (38%) from the first cohort and 64 patients (26%) in the second one. Of positive MRIs, most lesions were categorized as PI-RADS 5 (83%), followed by 4 (60%) and 3 (12%). The complications post-procedure were the same for both groups. MpMRI ± TB proved to be superior to SB, diagnosing less insignificant PCa, and avoiding a quarter of unnecessary biopsies [54].

A recent study published by Andres Labra et al. in 2020 investigated a cohort of 122 patients with mpMRI suspicion of prostate cancer undergoing MRI-TRUS fusion biopsy and SB biopsy. The positive results for Gleason 6 score were 56% (MRI-US fusion) vs. 48%(SB), representing only a small difference (*p* = 0.049), but for Gleason score of 7 and above, the difference became larger, 65% vs. 46% (*p* < 0.001). The fusion group scored better for patients with negative prior biopsies (48.7% vs. 38.5%, *p* < 0.13) and for patients with difficult lesions (anterior, transition, central zone) (68% vs. 27%, *p* < 0.001). SB missed 20% of csPCA identified by MRI-TRUS biopsy. The mpMRI PI-RADS scores of 3, 4, 5, identified cancer in 36.7%, 72.1% respectively in 90.3% of cases, in concordance with the pathological results. The authors state that if collecting only two cores from a targeted zone, one can omit 25% of csPCa [55].

MRI should be implemented for all patients considered for prostate biopsy. Bp-MRI is cheaper and faster than mp-MRI, and lacks complications related to contrast media. MRI-fusion biopsy seems to gain acceptance, but targeted biopsy alone is not the way, because in some cases, systematic biopsy identified 3.9% more csPCa and 6.8% PCa that were invisible to the MRI. In contrast, TB identified 12.1% PCa cases and 12.9% csPCA that SB omitted [56].

The NPV was 88% in detecting clinically significant PCa, which means that mp-MRI can overlook 12% of prostate cancers [57].

Another study published by Hashim U Ahmed et al. in 2020 included 576 men in a trial, that underwent mpMRI and afterward template prostate mapping (TPM) (with 5 mm distance sampling) biopsy and TRUS biopsy, each one blinded from the other. TPM detected 71% PCa, 40% csPCa, missed 2% (13 cases) csPCa diagnosed by TRUS. MpMRI showed 93% sensitivity, 41% specificity, NPV 89% and positive predictive value (PPV) 51%. Of 158 (27%) patients with negative MRI, 17 had csPCa(GS 3+4), detected at TPM biopsy. TRUS biopsy identified 452 insignificant PCa, of which 119 cases proved to be csPCa on TPM biopsy. If mpMRI is performed before the biopsy, about 25% of patients will avoid unnecessary biopsies, and it will improve the detection of csPCA and reduce the overdiagnosis and detection of clinically insignificant PCa. TRUS was performed after TPM, and this might contribute to the poor accuracy of TRUS. Since each test was blind, they could not evaluate the utility of MRI targeted prostate biopsies [58].

MpMRI did not prove an upgrade of PCa compared with SB in AS at two years follow-up in the ASIST trial, although MRI had a higher sensitivity for diagnosing csPCa (93% vs. 48%, p.0.0001). A lower upgrading rate suggests that MRI TB aimed at the lesion from the start and the 50% reduction in AS failure, reinforcing the role of MRI in AS management. The study was conducted before the introduction of PI-RADS v2 [58].

When mpMRI was compared with mpUS, on 82 patients with PSA, freePSA, density, and velocity above normal levels, mpMRI detected 54 lesions, of which 44 proved to be PCa (at biopsy), showing a sensitivity of 91% an specificity of 66%, in contrast to mpUS, of which transrectal elastography (TRES) proved the best sensitivity (69%), specificity (44%), and contrast-enhanced ultrasound (CEUS) showed a sensitivity of only 40% but a specificity of 97%. The authors concluded that the two investigations combined could help maximize the accuracy of prostate biopsy [59].

A systematic review conducted by Liang Zhen et al. in 2019 carried out between 2007 and 2017 included 29 studies of mpMRI, and found a pooled sensitivity of 0.87 and specificity of 0.68. Comparing MRI 1.5T with 3T, it seems that the 3T has higher sensitivity. Biparametric MRI versus multiparametric MRI showed similar sensitivity and specificity with the cost of higher signal heterogeneity and higher risks of artifacts [60].

In patients with PSA levels above 10 ng/mL and positive DRE, mpMRI brings a negligible benefit for diagnosing PCa. In general, mpMRI could reduce the number of unnecessary biopsies by 25% and increase the identification of High Grade (HG) PCa by up to 28% [61].

When comparing mpMRI with Prostate Cancer Antigen 3 (PCA3) and PHI for rebiopsy, mpMRI proved a higher accuracy in diagnosing PCa [62].

In the same context, Marloes van der Leest et al. conducted a study in 2018, and performed a head to head comparison between MRI followed by MRI guided biopsy vs. MRI and TRUS biopsy, and found that if patients with only PI-RADS 3–5 lesions would undergo biopsy, about 49% of men would be spared, with 3% missed csPCas. All the patients in this study underwent transrectal ultrasound-guided biopsy (TRUSGB), but only patients with PI-RADS 3–5 lesions also underwent “in bore” magnetic resonance-guided biopsy (MRGB). A percent of 4% of clinically significant prostate cancers were MRI nonsuspicious and identified by TRUSGB. Using only the positive MRIs when deciding to undergo biopsy could diagnose less insignificant PCas (25% TRUSGB versus 18% MRGB), and lower the complications rates [63].

MpMRI has established its role in the diagnosis process of PCa in active surveillance, and it also showed superiority over other markers (PSA, fPSA, PHI, PCA3) [14,62]. Recent studies show that bpMRI has similar results but is faster and cheaper. The main disadvantages are represented by the costs and the learning curve. It is not a perfect tool, and we consider that the direction of research would be towards combined tests that can also lower the economic burden and fasten the diagnosis process until a definitive decision.

### 4.2. Mp-US (Multiparametric Ultrasound)

Traditional TRUS can be improved with the addition of Tissue Harmonic Imaging and spatial compound imaging. Micro-ultrasonography (MicroUS) is referred to as frequencies of 14–29 MHz, which can offer a spatial resolution of 50–70 μm, with an up to 94% increase in PCa detection rate and AUC of 0.60–0.80. In this context, multicenter trials are necessary to confirm this new imaging device [64].

Strain elastography (SE) is a procedure where the prostate is compressed and decompressed with the transrectal transducer. It provides a color-coded image, with the less elastic zones colored in blue. It is not PCa specific, and the results for the moment are confounding [64].

In contrast to SE, shear-wave elastography (SWE), is another method of identifying stiff zones. Compression and decompression must be avoided; the prostate is color-coded, soft normal tissue with blue color and suspicious zones in red. In the case of false-negative MRI, SWE can find PCa in the peripheral zone, in 2/3 cases. Two meta-analyses found that SWE had an 83–86% sensitivity and 85–89% specificity for detecting PCa, and AUC of 0.94. It can distinguish peripheral nodules from BPH or macro-calcifications, and it can estimate Gleason score from stiffness. SWE is not powerful enough to exclude PCa without a prostate biopsy [64].

Both SE and SWE present limitations when facing large prostate glands; not all stiff lesions are cancers and not all cancers present stiffness [64].

CEUS is a technique in which microbubbles are injected via the bloodstream and act as an ultrasound contrast agent. However, it does not enter the urinary collecting system or interstitial space, and it is not contraindicated in renal insufficiency or urinary obstruction and has a low risk of an anaphylactic reaction (0.014%) [65]. It can identify vascular and microvascular architecture or irregularities around PCa. CEUS can enhance Doppler sensitivity from 54% to 93% and specificity from 79% to 87% in diagnosing hypervascular PCa nodules [64]. In a large prospective study, with 1024 patients, CEUS helped identify 20.5% more csPCa at biopsy, including 15.6% PCa missed by standard biopsy [65].

Mp-US is a concept derived from mpMRI, which includes improved B mode, vascular imaging, elastography, perfusion imaging, and volumetric imaging. It may include micro-Doppler elastography and CEUS. Its performance could be comparable to that of mpMRI [64].

The prostate risk identification-micro ultrasound (PRI-MUS) risk score represents a protocol that standardizes suspicious prostate lesion on TRUS with Micro-US (29 MHz). The Score ranges from 1–5, very low risk, some risk, intermediate-risk, significant risk, and very high risk. The authors agree that combining this protocol with multiparametric features can enhance the US power in diagnosing PCa, especially csPCa [66].

The initial experience at Cleveland Clinic with MicroUS, PRI MUS protocol, on 67 patients (38 PCa patients diagnosed), found that using the MicroUS after TRUS biopsy changed the diagnosis for eight (21%) patients and found six (26%) cases of csPCa that TRUS missed. In contrast, TRUS found seven PCa cases missed by MicroUS. A total of 19 patients underwent mpMRI targeted biopsy immediately after US biopsy (TRUS and MicroUS), and 10 out of those 19 patients were confirmed positive for PCa. Of all three techniques, MicroUS found two subjects positive for PCa where mpMRI and systematic biopsy were negative. In this study, the TRUS detection rate was 44%. Still, adding the Micro-US it raised to 56.7% and brought a relative improvement of 26.7% increase in diagnosing PCa. There was no statistical difference of added value to the diagnosis between mpMRI and MicroUS [67].

If patients are unfit to undergo mp-MRI for different reasons (pacemaker, claustrophobia, etc.) [68], CEUS proves to be a safe and cost-efficient alternative for most patients. In a prospective study conducted by Zhu Yunkai et al. in 2018, on 1024 patients, 378 were histologically positive for PCa. CEUS-TB (targeted biopsy) provided 27 cases of ISUP upgrade compared with SB, and longer cancer cores. It diagnosed more significant PCa in 67 cases (28.7% vs. 25.3%) and only ten more cases of insignificant PCa. In contrast, SB diagnosed 40 cases more insignificant than CEUS-TB, but it also found 32 cases of significant PCa missed by the other one. CEUS provided promising results for patients with PSA levels ≤ 10 ng/mL, and prostate volumes of 30–60 mL [65].

Using a 7 MHz probe, and color Doppler ultrasound (CDUS) and spectacular micro-vascular imaging (SMI), 74/119 (62.2%) of patients were diagnosed with PCa (biopsy confirmed). Of these, SMI proved to be superior in identifying blood vessels, and it identified abnormal vascularity in 97.3% (72/74 patients) of cases vs. 90.5% (67/74 patients) (CDUS). The abundance of vascularity demonstrated a positive correlation between SMI, CDUS, and Gleason score. SMI-TB detected 89.2% (66/74) PCa cases vs. SB with 58.1% (43/74) cases, SMI-TB also identified 21 PCa patients missed by SB, and five patients underdiagnosed by SB. In contrast, when diagnosing Gleason 6, SB proved superiority in 10.8% (8/74 patients) vs. 1.4% (1/74 patients) of cases [69].

In a study conducted by Giovanni Lughezzani et al., in 2018, 104 patients underwent mpMRI and then MicroUS. The ultrasound urologist was blinded to the MRI results. From a total of 104 patients, mpMRI identified 21 insignificant PCa cases, 35 csPCa, and 48 benign cases. At the same time, MicroUS suspected 83 patients from a total of 104, 15 of them were found to be insignificant PCa, 33 csPCa, and 35 benign cases. Of the 21 patients with no suspect lesions, 13 were benign, six insignificant PCa, and two csPCas. MicroUS showed a sensitivity of 94%, specificity of 28% in finding csPCa. Of the 138 mpMRI targeted lesions, 48 (33%) were positive, 32 (23%) were csPCa. MicroUS targeted 117 lesions, of which 39 (33%) were positive. Of these, 33 (28%) were csPCa. The two investigations were concordant in 61 (45%) of 136 targeted lesions [70].

One of the advantages of ultrasound over MRI is that the investigation usually is faster, an US scan takes about 5 to 10 min and the MRI 25 min. Besides that, the US has the advantage that it is portable [71].

A multicenter prospective study published in 2020, where uro-radiologist experts on mpMRI and expert urologists on ultrasound examinations with at least five years of experience each tried to compare the results of mpMRI and micro-US in detecting PCa. For identifying PCa GG ≥ 2, micro-US had a sensitivity of 94% vs. mpMRI with 93%, (*p* < 0.03) specificity of 22% vs. 23% (*p* = 0.01 for non-inferiority). Micro-US showed similar specificity and sensitivity compared with the mpMRI, and the authors stated that it represents a strong alternative, is easier to use, and is familiar to the urologic community [68].

MpUS represents a novel technique, unique features with a lot of interest by urologists. It has shown promising results; some studies even achieved similar sensitivities and specificities with mpMRI. It has the advantage of being a fast procedure, being easy to do, but being a novel technique and the learning curve represent disadvantages.

## 5. Combined Tests

From the biomarkers and techniques presented above, none of them are ideal, each one presents advantages and disadvantages. In the following chapter, we present studies that combined the above-mentioned tools in order to be more effective in the PCa diagnosis process.

Combining the 4Kscore and mp-MRI provides more clinical information and leads to fewer biopsies with similar clinical PCa diagnoses compared to each investigation alone. If the 4Kscore is of low risk (<5%), it can even avoid MRI, or if it is of high risk (>23), the patient can undergo biopsy without MRI. Mp-MRI was useful for intermediate-risk categories [57]. This association between tests allowed 51% (151 patients) of men to avoid unnecessary biopsies from a total of 300 who were evaluated. Among the 149 patients who underwent biopsies, 73 (49%) had PCa, of whom 49 (33%) had Gleason Score 7. The 4Kscore associated with mpMRI provided an AUC of 0.82 (0.75–0.89) in contrast to each test individually, 4Kscore AUC 0.70(0.62–0.79), mpMRI AUC 0.74 (0.66–0.81) *p* = 0.001 [72].

The 4Kscore (PSA based test) and Select MDx (non-PSA based test) were combined to aid in deciding to perform the prostate biopsy or not, but the tests were discordant in 45.6% of patients. Compared to each other, the AUC was 0.830 for 4Kscore and 0.672 for SelectMDx (*p* = 0.036) in detecting clinically significant PCa65 [73]. Select MDx is a gene panel that consists of HOXC6, DLX1, TDRD1 genes, and it showed an AUC of 0.86 for detecting csPCa. It can reduce unnecessary biopsies by 42% while missing only 2% of csPCa [15].

In order to better diagnose PCa, in 2019, Ugo Giovanni Falagario et al. proposed three strategies to combine 4Kscore, mpMRI, and PSAD. The first strategy starts with the evaluation of the 4K value. If it was > 7.5 (intermediate or high risk), they conducted a mpMRI. If the mpMRI was positive, the patient underwent biopsy, but if it was negative, and 4Kscore was above 7.5, but under 18 (intermediate risk), the patient went under clinical follow-up. The second strategy was similar, but the mpMRI was conducted first. The third strategy was based on mpMRI, and if positive, then PSAD would be calculated (cutoff <0.10 ng/mL/cm^3^). The first and second strategy missed 2.7% of csPCa and the third only 1.3%. In conclusion, 4Kscore lowered the number of mpMRIs, without missing a lot of csPCa. In general, it is a fast and cheap test, but if added after mpMRI, it can raise the total costs [72].

Laura Wiemer et al. conducted a study published in 2020, where patients underwent mpMRI (PI-RADS V2); after that, they were subjected to blinded Micro-US (PRI-MUS), and finally standard biopsies (12 cores) were performed. An extra two cores were taken from each PCa suspect lesion visualized on MRI or MicroUS. The latter identified 17% (27/159 patients) more PCas that had MRI negative targeted biopsies, and 20 of those patients were csPCa. From a total of 159 cases, in 58% (46% were csPCa) the MRI showed the same results as MicroUS. In 26% cases, MRI showed higher grading, and in 16% cases, micro-US showed higher grading, adding value in detecting csPCa. If standard biopsies had been eliminated, both methods would have missed only 3% of csPCa [74].

**Figure 1:**
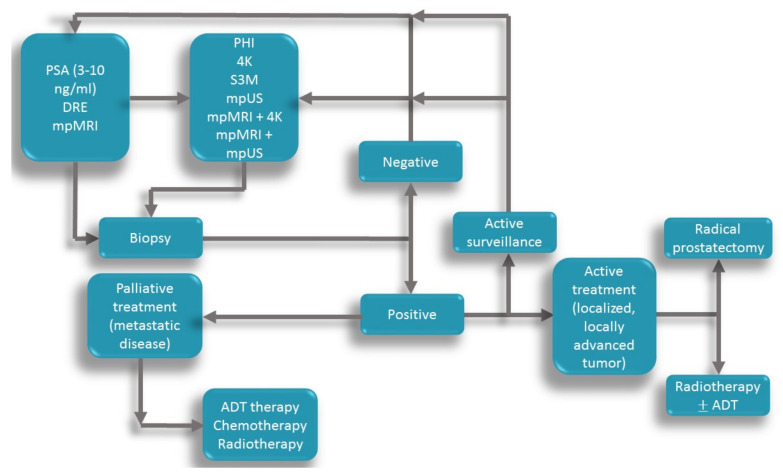
The pathway for diagnosis and treatment of prostate cancer. The diagram represents the patient’s flow from the moment he presents a suspicious PSA level. First, direct rectal examination (DRE) and mpMRI are part of the standard of care. Before the biopsy, the patient should perform one of the tests that will further inform about the patient’s risk of Pca and only then proceed with the biopsy. If it is negative, the patient returns to the diagnostic process, but if the biopsy is positive, the patient can enter active surveillance (for low-risk cancers) and undergo tests periodically, or if the cancer is intermediate- or high-risk, the patient will suffer curative treatment (radical prostatectomy of radiotherapy). If curative treatment is not an option, the patient (with metastatic disease) will undergo palliative care (ADT—androgen deprivation therapy, chemotherapy, radiotherapy).

The classic diagnosis pathway includes PSA DRE and mpMRI, which leads straight to the prostate biopsy. Still, if the novel biomarkers would be performed before the biopsy, it would bet triage the patients that this invasive maneuver. The same thing is valid for previous negative biopsies or for patients under active surveillance. Figure 1 is a graphical representation of diagnosis and treatment strategies applied in the PCa investigation.

## 6. Other Perspectives

The biomarkers that we consider of interest are described above and summarized in Table 2. Still, there are also other biomarkers to consider in the pre-diagnostic stage like PCA3, MiPS (from urine and blood), SelectMDX, and ExoDx prostate IntelliScore which are urine tests, or ConfirmMDx, which is a tissue test used in the case of negative biopsy and post-biopsy, and once the diagnosis is established, biomarkers like Oncotype Dx, ProMark, Prolaris, and Decipher have a prognostic role [75].

From the imaging point of view, mpMRI has established its role in the diagnosis of PCa, and US represents a technique that is catching up, but these two are the only ones used in cancer diagnostics. Another technique that proved its usefulness is the positron emission tomography-computer tomography (PET-CT) for staging and metastasis identification. In PCa, the tracers used are Choline and Gallium 68 prostatic specific membrane antigen (^68^Ga-PSMA), the latter proving its superiority in identifying metabolically active metastasis outside the prostate, it identified metastasis in 48% of patients that had negative choline PET-CTs. It can identify lymph nodes as small as 8 mm. The prostate or prostate bed (after radical prostatectomy) is difficult to evaluate because of the tracer accumulates in the urine, especially in the bladder and urethra [77,78].

MicroRNAs (MIRNAs) represent small (21–25 nucleotides) non-coding RNAs that regulate gene and protein expression. They have tumor repressor or oncogenic roles, are tissue-specific, and can be found in blood, urine, or tissue. Their levels can be modified in all stages of PCa, from incipient tumors to metastatic states. These molecules look promising but further studies for validations are needed [79,80].

Standard Risk Calculators such as ERSPC RPCRC, PCPT (Prostate Cancer Prevention Trial) model, and the Prostate Class model, are validated and can be used to stratify csPCa better. Novel ones like ERSPC RPCRC3 include new biomarkers (PHI) and showed better detection of PCa with an AUC of 0.75 [15].

In a scenario where ERSPC RPCRC is performed, if the result is positive, the MRI would have been performed, and this strategy could reduce the MRIs and prostate biopsies by 37% while missing about 4% of csPCa [81].

Another essential aspect that concerns physicians is economic burden. In the US, for men between 55–69, a low frequency (4 years) active surveillance could be cost-efficient, at a threshold of USD 10,000, but it loses its cost efficiency if there are shorter screening intervals or if there is a need for immediate treatment [24].

In the screening programs for Chinese men, adopting a PHI strategy proved to be more cost-effective than the PSA strategy [82].

MpMRI and biopsy could cost around GBP 965/patient, but using PHI ≥ 30 before mpMRI, reduced costs with about 20% (GBP 774/patient) [41].

Ultrasound scanners cost about GBP 10,000 [71], but they are cheaper in comparison with an MRI system. Another problem is the training of the specialists, but this is valid for both techniques.

In Australia, the biopsy process median costs were about 2711 Australian Dollars if the transrectal technique is conducted, and 3441 Australian Dollars for the transperineal approach. The introduction of mpMRI as a triage agent allowed them to save 784 Australian Dollars per patient and reduced the number of biopsies by 47% [27].

In the United States, Niranjan et al. developed a model in which patients with PSA >3 ng/mL would undergo TRUSBx as part of the standard of care for diagnosing PCa or they would have performed one of three tests (PHI, 4Kscore, SelectMDx). The standard of care cost was about USD 3800 per patient using PHI or SelectMDx as part of the process, lowering the charges, but on the other hand, 4Kscore proved to be more expensive [83].

In Table 3 are represented the economic impact of the data mentioned above.

## 7. Conclusions

The era in which TRUSbx was performed based only on PSA values is gone. PSA is slowly becoming an indicator for further investigations, not a test to determine if a patient should proceed to biopsy or not.

PHI, 4Kscore, STHLM3, MRI and mpUS showed superior sensitivity and specificity over PSA in the diagnosing process, active surveillance, discrimination between malign and benign, and between indolent and aggressive tumors. In addition, recently merged tests proved superiority over singular ones, and they can provide a smooth patient flow that will not overwhelm the system.

These tests were investigated for a short period of time; prospective studies are needed to determine if they play a role in survival, metastatic advancement, or cancer-specific mortality. To date, there is no test available that is accurate enough to skip tissue sampling. The validation of these biomarkers needs to be confirmed on large cohorts because statistical difference does not necessarily mean clinical difference. In summary, it is evident that biomarkers come with a benefit in the decision making of prostate cancer diagnosis, and moreover, most of them are easily accessible.

Finally, in the future, biomarkers and imaging techniques for prostate cancer diagnosis will have to be used in an interconnected routine rather than in a competitive one, in order to determine the best patient selection.

## Figures and Tables

**Table 1:** Prostate-specific antigen (PSA)-based screening recommendations.

Screening Recommendation Associations	Recommendations
AUA (American Association of Urology) [30]	PSA screening for men 55–70 years old Urinary, serum biomarkers, imaging, Risk Calculators can be used for men with a suspicious PSA level Screening at 2 years or more can be applied to reduce the harm of screening No screening if life expectancy is <15 years old, or men >70 years old
EAU (European Association of Urology) [8]	PSA screening for men over 50-year-old or over 45 if they had a family history of PCa, African descent or over 40 if carrying BRCA2 mutations Men with PSA level >1 ng/mL at 40-year-old or >2 ng/mL at 60-year-old are at risk Men with PSA 2–10ng/mL and normal DRE, prior to biopsy, use additional tests (PCA3, PHI, 4Kscore, Kallikreins, TMPRSS2-ERG or Risk Calculators, Imaging (mpMRI) PSA screening every 2 years for those at risk
ESMO (European Society of Medical Oncology) [31]	Subclinical PCa is common in men >50-year-old PSA screening for men 55–70 years old PSA level >1 ng/mL for men at 40 years old and >2 ng/mL for men at 60-year-old represents a risk Early PSA screening for men >50 years old, >45 with a family history of PCa, African-American, and BRCA 1/2 carriers Do not test if life expectancy is <10 years Use Risk Calculators or mpMRI before biopsy
ACS (American Cancer Society) [32]	Informed PSA screening for men >50 years old, >45 if African-American or men with first-degree relative diagnosed with PCa (under the age 65), >40 years old if they have more than one first degree relative with PCa Early screening for men with PSA level 2.5 ng/mL If the biopsy is negative, additional tests can help (PHI, 4Kscore, PCa3, ConfirmMDx)

**Table 2:** Representative studies for recent advances in diagnose of PCa (inclusion criteria: study design and number of patients).

Marker/Technique	Number of Patients	Study Design	Cancer Detection	Other Details
PHI [41]	545	Prospective, multicentric study	PHI AUC 0.82 PSAD AUC 0.79 PSA AUC 0.70	If MRI is negative, PHI AUC for positive PCa 0.78 If PHI ≥ 30, 35% of MRIs could be avoided Spared 40% of biopsies Missed 8% PCas
4Kscore [46]	2 224 PCa 2 230 controls	Prospective, multicentric, case-control, multiethnic	4K AUC 0.782 PSA+fPSA AUC 0.739 PSA AUC 0.685	The AUCs represent the identification of aggressive PCas 4kscore can accurately differentiate between benign and malign cases, indolent and aggressive tumors
S3M [50]	59 149	Prospective, population baased, diagnostic trial	S3M AUC 0.75 PSA AUC 0.58	34% of biopsies spared
MRI [76]	576	Prospective, multicenter, paired-cohort	sensitivity 93%, specificity 41%	Could avoid 27% of biopsies TRUS directed by MRI, could diagnose 18% more csPCas
mpUS [68]	1040	Prospective, multicenter	mpUS sensitivity 94%, specificity 22%	Results were similar with mpMRI sensitivity 93%, specificity 23%
4Kscore, MRI, PSAD [72]	266	Retrospective, unicentric	4Kscore and MRI if 4K > 7.5	4kscore followed by MRI if 4K > 7.5 and biopsy if MRI is positive or biopsy if MRI negative and 4K > 18

**Table 3:** Economic aspects of different tests around the world.

Country	Test	Costs
Germany [34]	PHID	100 EUR
China, Hong Kong [82]	PHI	370 USD
US [15]	PHI	80 USD
	4Kscore	500 USD
	PCA 3	300 USD
	MiPS	700 USD
	SelectMDx	300 USD
	ERSPC RPCRC Risk Calculator	0
	PCPT Risk Calculator	0
	MRI	1000 USD
Europe [15]	MRI	300–500 EUR
UK [71]	US Scanner	35,000–150,000 USD
	MRI machine	Approx. 3 million USD
	MRI + biopsy	965 GBP

## Abbreviations

4K	4 kalikrein score
^68^ Ga-PET-CT	Gallium 68 positron emission tomography- computer tomography
ACS	American Cancer Society
ADC	apparent diffusion coefficient
AS	active surveillance
AUA	American Urology Association
AUC	area under the curve
BMI	body mass index
BpMRI	bi parametric magnetic resonance imaging
BPH	benign prostatic hyperplasia
BRCA	breast cancer gene
CAP	The Cluster Randomized Trial of PSA Testing for Prostate Cancer
CDUS	color Doppler ultrasound
CEUS	contrast-enhanced ultrasound
csPCa	clinically significant PCa
DCE	dynamic contrast enhancement
DRE	digital rectal examination
DWI	diffusion-weighted imaging
EAU	European Association of Urology
ERSPC	European Randomized Study of Screening for Prostate Cancer trial
ESMO	European Society of Medical Oncology
FDA	Food and Drug Administration
fPSA	freePSA
GG	Gleason Grade
HG	high grade
ISUP	International Society of Urological Pathology
MIC1	macrophage inhibitory cytokine-1
MicroRNAs	MiRNA
Mp MRI	multiparametric magnetic resonance imaging
Mp US	multiparametric ultrasound
MRGB	magnetic resonance guided biopsy
MSMB	microseminoprotein beta
NPV	negative predictive value
PCa	prostate cancer
PCA3	prostate cancer antigen 3
PCPT	Prostate Cancer Prevention Trial
PET-CT	positron emission tomography- computer tomography
PHID	prostate health index density
PHI	prostate health index
PI-RADS	Prostate Imaging–Reporting and Data System
PLCO	The Prostate, Lung, Colorectal and Ovarian
PPV	positive predictive value
PRI-MUS	Prostate risk identification-micro ultrasound
PSA	prostatic specific antigen
PSAD	PSA density
ROC	receiver operating characteristics
RPCRC	Rotterdam Prostate Cancer Risk Calculator
SB	systematic biopsy
SE	strain elastography
SMI	spectacular micro-vascular imaging
STHLM3 (S3M)	Stockholm 3 test
SWE	shear wave elastography
T2W	T2 weighted image
TB	targeted biopsy
TP	transperineal
TPM	template prostate mapping
tPSA	Total PSA
TR	transrectal
TRES	transrectal elastography
TRUS	TransRectal UltraSound
TRUSGB	transrectal ultrasound-guided biopsy
